# Informatics-Based Psychotherapeutic and Psychiatric Interventions in Dermatology: Scoping Review of Impacts on Skin Disease Severity and Mental Health Outcomes

**DOI:** 10.2196/82096

**Published:** 2026-06-10

**Authors:** Caroline Lamarre, Jeffrey Chivinski, Alexandre Hudon

**Affiliations:** 1 Department of Medicine, Faculty of Medicine, Université de Montréal, Montréal, QC, Canada; 2 Beacon Dermatology, Calgary, AB, Canada; 3 Department of Psychiatry and Addictology, Faculty of Medicine, Université de Montréal, 2900 Boulevard Édouard-Montpetit, Montréal, QC, H3T 1J4, Canada, 1 5142514000; 4 Department of Psychiatry, Institut universitaire en santé mentale de Montréal, Montréal, QC, Canada; 5 Department of Psychiatry, Institut national de psychiatrie légale Philippe-Pinel, Montréal, QC, Canada; 6 Centre de recherche de l'Institut universitaire en santé mentale de Montréal, Montréal, QC, Canada; 7 Centre de recherche en pédagogie de la santé, Montréal, QC, Canada

**Keywords:** psychodermatology, digital health, cognitive behavioral therapy, teledermatology, mental health, dermatologic conditions, mobile health apps, digital therapeutics, psychiatric comorbidity, scoping review

## Abstract

**Background:**

Chronic dermatologic conditions such as psoriasis, atopic dermatitis, and hidradenitis suppurativa are associated with a high burden of psychiatric comorbidities, including depression, anxiety, and suicidality. Despite growing awareness of the psychosocial impact of skin diseases, mental health needs remain underaddressed in dermatologic care. Digital technologies (including teledermatology, mobile health apps, and internet-delivered psychotherapies) offer promising avenues for integrating psychotherapeutic and psychiatric interventions into dermatology. However, the scope, effectiveness, and implementation of such informatics-based approaches remain poorly mapped in the literature.

**Objective:**

This scoping review aimed to systematically identify, categorize, and synthesize studies on digital psychotherapeutic and psychiatric interventions targeting patients with dermatological conditions, with a focus on clinical, mental health, and implementation outcomes.

**Methods:**

Following the PRISMA-ScR (Preferred Reporting Items for Systematic Reviews and Meta-Analyses extension for Scoping Reviews) guidelines, we conducted a comprehensive search across 5 databases (MEDLINE, Embase, Web of Science, PsycINFO, and Google Scholar) for articles published up to March 2025. Studies were included if they involved patients with dermatologic conditions and assessed interventions that combined a digital informatics component (eg, telehealth, apps, artificial intelligence, virtual platforms) with a psychotherapeutic or psychiatric element (eg, cognitive behavioral therapy [CBT], mindfulness, consult-liaison psychiatry). Eligible study designs included clinical trials, observational studies, and mixed methods research. Data were extracted systematically, and methodological quality was assessed using JBI tools.

**Results:**

Out of 15,176 records identified, 11 studies met the inclusion criteria. Most interventions targeted psoriasis (9/11) and used asynchronous digital platforms such as internet-based CBT and mobile apps. Across studies, dropout rates ranged from 10% to 76%. Improvements in dermatologic quality of life were reported in 6 of 11 studies, with statistically significant reductions in depression and anxiety observed in multiple trials (eg, internet-based CBT and mindfulness-based interventions), alongside reductions in psoriasis severity (Psoriasis Area and Severity Index) and itch intensity in randomized controlled trials. Intervention duration ranged from single-session virtual reality exposure to 8‐ to 12-week structured programs. However, long-term outcomes beyond 3 to 12 months were rarely assessed, and reporting of sociodemographic variables and equity-related factors was limited.

**Conclusions:**

Informatics-based psychotherapeutic and psychiatric interventions represent a promising frontier in psychodermatology, with early evidence suggesting feasibility and potential clinical benefit. Digital platforms may expand access to mental health support and improve holistic care for patients with dermatologic conditions. However, significant gaps remain in terms of equity, long-term effectiveness, integration into clinical workflows, and adaptation for diverse populations. Future research should focus on rigorous, inclusive trials and the development of hybrid models that blend digital and face-to-face care to ensure sustainable and equitable impact.

## Introduction

### Dermatologic Issues and Mental Health

Chronic dermatologic conditions, such as psoriasis, atopic dermatitis (AD), acne, vitiligo, and hidradenitis suppurativa (HS), are among the most psychologically burdensome medical conditions and impose a substantial, multifaceted impact on mental health. Not only do these cutaneous diseases cause visible disfigurement, discomfort, and functional and social impairment, but they are also associated with a high prevalence of various psychiatric comorbidities. Chronic symptoms like pain and pruritus associated with the relapsing nature of many skin conditions often mediate these psychological effects [1]. Large-scale studies have found that around 10% of patients with dermatologic conditions meet the criteria for clinical depression and around 17% for clinical anxiety, with about 12% to 13% reporting suicidal ideation [1]. These rates are roughly 2 to 3 times higher than in the general population, underscoring how common serious psychological comorbidities (eg, depression, anxiety, social anxiety or phobia, and suicidal thoughts) significantly add to patients’ overall disease burden [1]. For instance, psoriasis and AD are each associated with notably elevated risks of new-onset depression and anxiety. A recent UK cohort study reported that adults diagnosed with psoriasis or AD had a 10% to 20% greater hazard of developing depression or anxiety within the first year, compared to matched individuals without skin disease [2]. In particular, patients with more severe skin involvement (such as extensive psoriasis) show even higher relative risks of depression and other psychiatric sequelae [2].

Psychiatric morbidity appears especially pronounced in HS, which research indicates is accompanied by a high prevalence of depression and anxiety, as well as increased rates of substance use disorders and even serious mental illnesses such as psychosis or bipolar disorder [3]. This higher prevalence is due to a combination of biopsychosocial factors, such as chronic pain and physical discomfort secondary to malodor and constant drainage. Many patients develop social withdrawal and avoidance behaviors out of fear of stigmatization. As the disease often affects intertriginous and genital areas, patients experience an impact on their sexual health and relationship difficulties. A lack of cure or repeated ineffective treatments leads to hopelessness and despair over time. Most alarmingly, patients with HS face a dramatically elevated risk of suicidality. For example, epidemiologic studies have found that suicide is roughly twice as likely in HS sufferers compared to those without HS [3]. Such findings illustrate that chronic inflammatory skin diseases carry not only physical burdens but also profound psychological and social challenges for patients. This underlines the need for integrated, multidisciplinary approaches to care for these patients.

Despite this heavy psychosocial burden, patients’ mental health needs are often overlooked in dermatologic care. In 1 large survey of over 7200 patients with inflammatory dermatoses, two-thirds reported high stress levels, yet fewer than 15% had ever been offered any psychological support [4]. Dermatology clinics traditionally focus on cutaneous findings, and many patients never receive screening or referral for underlying depression, anxiety, or other psychological issues related to their skin condition [1]. These unmet needs can be due to under-recognition by care providers, limited access to mental health care like psychology or psychiatry, or simply the stigma associated with mental illness. Dermatologists often lack the time or referral pathways to manage the psychologic comorbidities. This gap in care means that a significant portion of patients with dermatologic disorders endure unaddressed emotional distress, which can, in turn, exacerbate disease outcomes. Furthermore, this can lead to poor adherence to medical treatments and a worsening quality of life. Consequently, there is a growing consensus that integrating mental health screening and psychotherapeutic support into routine dermatologic practice is critical for the optimal management of chronic skin diseases. Experts advocate for the regular use of mental health questionnaires (eg, Patient Health Questionnaire-2 for depression and Generalized Anxiety Disorder-7 for anxiety) in dermatology settings and prompt referral to psychiatry or psychology when needed [5]. Studies suggest that comprehensive care, combining dermatologic treatment with interventions, such as cognitive behavioral therapy (CBT), stress management techniques, or support groups, can improve patients’ quality of life and potentially even their skin disease severity [6]. By recognizing and addressing the psychological comorbidities of chronic dermatoses, clinicians can substantially reduce the overall disease burden and improve long-term outcomes for these patients.

### Psychotherapeutic Interventions and the Emerging Role of Digital Technologies

Digital platforms (including teledermatology, mobile health [mHealth] apps, artificial intelligence [AI]–supported therapeutics, and virtual assistants) are reshaping dermatologic care and have emerged as innovative ways to provide and deliver therapeutic interventions to patients with chronic medical conditions. Teledermatology (via store-and-forward images or live video) enables remote diagnosis and management of chronic inflammatory skin diseases with high reliability. It can significantly shorten waiting times for patients while maintaining diagnostic accuracy comparable to in-person visits [7]. For example, 1 study on patients with chronic skin conditions found that live-video teledermatology achieved 96% diagnostic concordance with face-to-face consultations and reduced consultation turnaround time by 90% [8]. This translates to faster treatment and greatly improved access for patients who might otherwise face long delays or travel barriers due to living in remote areas. In addition to bypassing geographic barriers, teledermatology can help reduce stigma or anxiety associated with in-person therapy and is more flexible. mHealth apps offer accessible tools for patient self-monitoring, education, and behavior change. Dermatology apps can help patients track symptoms (for instance, logging eczema or psoriasis flares), adhere to treatment plans, and learn about skincare, thereby empowering them in day-to-day disease management [9]. Multiple scoring systems self-reported by patients can be integrated into these apps, for example: the Numeric Rating Scale for evaluating itch, or Skindex, which measures the emotional, symptomatic, and functional burden of skin diseases. Recent evidence confirms that such mHealth interventions improve patient outcomes: a 2024 meta-analysis showed that smartphone apps and telemonitoring significantly enhanced patients’ quality of life and self-management of AD, while also increasing patient satisfaction and engagement in care [10]. Meanwhile, AI-driven digital therapeutics and virtual assistants are being integrated into dermatology workflows to support both providers and patients. In a teledermatology program for psoriasis, adding a virtual assistant (chatbot) improved medication adherence and gave patients greater peace of mind by connecting them to their dermatologist; notably, participants’ Dermatology Life Quality Index (DLQI) scores improved with the chatbot intervention [11]. Such digital assistants can streamline triage, answer patient questions, and automate follow-ups, augmenting the efficiency of teledermatology services [11].

Importantly, these technologies do not just address skin health in isolation. They also impact mental health outcomes. Many chronic dermatoses carry a significant psychological burden (stress, low self-esteem, anxiety), so bridging dermatologic care with psychosocial support is crucial. Digital platforms are beginning to fill this need. For instance, an internet-based cognitive behavioral therapy (iCBT) program for patients with atopic eczema significantly reduced itching and insomnia while improving quality of life, with outcomes comparable to traditional therapist-led care [12]. This kind of online psychodermatologic intervention is valuable because access to in-person psychological services for patients with skin diseases is often limited [12]. In sum, teledermatology and related digital innovations are enhancing chronic skin disease management by improving convenience and access, supporting patient self-management, and addressing the psychosocial dimensions of dermatologic care in ways that were not previously possible. These interventions enhance the resilience and quality of life of patients with chronic skin diseases.

### Gaps in the Literature

Despite the growing promise of digital platforms in dermatologic and psychodermatologic care, their real-world implementation remains fraught with challenges. The presence of digital psychodermatology tools continues to be fragmented and sparse in clinical practice. Clinicians cite significant barriers related to workflow integration, including the time required for image capture and documentation; limited interoperability with electronic medical records; and medicolegal concerns surrounding data privacy, reimbursement models, and clinical liability [13,14]. From the patient perspective, digital literacy and technology acceptance remain key hurdles (particularly among older adults), whose comfort with telehealth interfaces is shaped by varying levels of support, familiarity, and personal values [15]. While digital tools such as teledermatology and online psychotherapeutic interventions have demonstrated improved access and high patient satisfaction, their clinical effectiveness remains inconsistent. Some models yield optimal outcomes only when used in conjunction with traditional in-person care [13,16]. Furthermore, many digital mental health interventions in dermatology, such as app-based CBT or stress management platforms, face high attrition rates and low long-term engagement, limiting their sustained impact on mental health outcomes [17]. These implementation and engagement challenges highlight the need for better-designed, clinically integrated, and user-centered digital solutions in psychodermatology.

### Objectives and Hypotheses

This scoping review aims to systematically map, categorize, and synthesize the scientific literature on informatics-enabled psychotherapeutic and psychiatric interventions designed to improve both dermatologic conditions and their associated mental health outcomes. It also seeks to address key gaps identified in the existing literature. Specifically, the objectives are to: (1) chart the landscape of digital platforms (such as teledermatology, mHealth apps, AI-based decision-support tools, iCBT modules, mindfulness-based digital programs, and e-psychiatry consult-liaison services) used therapeutically in dermatologic care; (2) profile the dermatologic diagnoses most frequently targeted and characterize the demographic and clinical features of the patient populations involved; (3) synthesize reported clinical, psychological, and quality-of-life outcomes, including dermatologic severity indices, itch and pain scores, validated mental health scales, and patient-reported outcomes; (4) analyze implementation models, including modes of delivery, duration, provider roles, integration into clinical care pathways, and equity-related considerations (eg, digital literacy, socioeconomic factors, and geographic access); (5) assess the methodological quality of included studies using validated critical appraisal tools to identify strengths, limitations, and risks of bias; and (6) identify knowledge gaps and research priorities to guide the future development, evaluation, and equitable implementation of digital psychodermatologic interventions.

## Methods

### Search Strategies

To comprehensively capture the current state of research on informatics-based psychotherapeutic and psychiatric interventions in dermatology, we conducted a structured scoping review of studies published up to March 2025. Searches were performed across 5 major electronic databases: MEDLINE (via PubMed), Web of Science, Embase, PsycINFO (via Ovid), and Google Scholar. The review followed the PRISMA-ScR (Preferred Reporting Items for Systematic Reviews and Meta-Analyses extension for Scoping Reviews) checklist (Checklist 1), which provides methodological guidance for mapping emerging fields with conceptual or methodological diversity [18].

The search strategy was developed iteratively, combining free-text keywords and controlled vocabulary (Medical Subject Headings and Emtree terms) to ensure broad yet relevant coverage. Terms related to dermatologic diagnoses (eg, “psoriasis,” “acne,” “atopic dermatitis,” “hidradenitis suppurativa”) were combined with those describing digital modalities (eg, “teledermatology,” “mobile health,” “artificial intelligence,” “digital therapeutics,” “virtual assistants”) and psychosocial or psychiatric constructs (eg, “depression,” “anxiety,” “psychotherapy,” “CBT,” and “mental health”). The objective was to capture literature at the intersection of digital tools and mental health outcomes in populations with dermatologic disorders. Search strategies were peer-reviewed internally and piloted for sensitivity before full execution. The scoping review was not registered.

All searches were conducted by the lead reviewer (CL), with results independently verified by a second reviewer (AH) to ensure consistency, accuracy, and completeness. No restrictions on study geography, clinical setting, or publication language were applied at the search stage, although inclusion criteria were applied during screening. Complete search algorithms for each database are provided in Multimedia Appendix 1.

### Study Eligibility

Studies were eligible for inclusion if they met all of the following criteria: (1) the population under investigation comprised children, adolescents, or adults with a clinically diagnosed chronic dermatologic disorder (eg, psoriasis, AD, acne, HS, vitiligo, alopecia areata, chronic urticaria, or other specified inflammatory or pigmentary skin diseases); (2) the study evaluated a therapeutic approach that incorporated both an informatics component (eg, teledermatology, mobile or e-health apps, web-based platforms, AI-driven tools, virtual reality [VR], chatbot technologies) and a psychotherapeutic or psychiatric element aimed at improving mental health or dermatologic outcomes. Acceptable psychological components included, but were not limited to, CBT, mindfulness-based interventions, psychoeducation, liaison psychiatry, or psychotropic medication management. Studies were eligible if they reported at least 1 skin-related outcome (eg, disease severity scores, itch, pain, or DLQI) or 1 mental health outcome (eg, anxiety, depression, psychological distress, or health-related quality of life). Eligible study designs encompassed randomized and nonrandomized controlled trials, quasi-experimental studies, cohort or cross-sectional analyses, mixed methods evaluations, and implementation studies with quantitative or qualitative outcome reporting.

Studies were excluded if they focused exclusively on diagnostic tools, image recognition systems, or data-capture technologies without a therapeutic or psychiatric objective. Similarly, studies assessing only pharmacological, surgical, or cosmetic interventions without an integrated digital or psychological component were not considered. Single-case reports with fewer than 5 participants, editorials, opinion articles, conference abstracts without full text, narrative reviews, and protocol papers lacking original data were also excluded. Additionally, studies involving only healthy volunteers, individuals without a dermatologic diagnosis (eg, cosmetic users), or interventions targeting sun protection, skin cancer screening, or esthetic counseling were not included. Only articles published in English or French were considered for review; gray literature and unpublished manuscripts were excluded.

### Data Extraction

A structured data extraction process was conducted using a predesigned coding framework in Microsoft Excel to ensure systematic and reproducible capture of study details. One reviewer (CL) performed the initial data extraction, and a second reviewer (AH) independently reviewed all entries to verify accuracy and consistency across studies. Discrepancies in interpretation or classification were resolved through discussion until consensus was reached.

Variables extracted encompassed bibliographic details (eg, author names, publication year, country of origin, and publication type), participant characteristics (eg, dermatologic diagnosis, sample size, age distribution, and gender), and intervention attributes (eg, program title, type of informatics platform, psychotherapeutic or psychiatric components, mode of delivery, and intervention duration). Study design parameters were also recorded, including methodological approach, setting, comparator groups, and factors related to health equity. Outcome measures included both clinical (eg, skin severity scores, itch or pain levels, DLQI) and psychological indicators (eg, symptoms of depression, anxiety, stress, or overall mental well-being), as well as implementation-related variables, such as adherence, usability, accessibility, and equity considerations. Where applicable, methodological quality and potential sources of bias were assessed using validated critical appraisal tools.

To enhance interpretability and align with digital health implementation frameworks, interventions were further categorized according to interaction modality and level of human involvement into three predefined groups: (1) *synchronous interventions*, defined as real-time interactions between patients and providers (eg, telepsychiatry, video consultations, live e-consults); (2) *asynchronous interventions*, defined as time-independent, user-initiated, or platform-delivered content without real-time clinician interaction (eg, mobile apps, web-based iCBT modules, educational platforms); and (3) *automated interventions*, defined as systems delivering therapeutic or support functions with minimal or no human involvement, often leveraging algorithmic or AI components (eg, chatbots, AI-assisted triage tools, VR environments). Classification was performed independently by 2 reviewers, and discrepancies were resolved by consensus.

### Quality Assessment

The methodological quality of all included studies was assessed using design-specific critical appraisal tools developed by the Joanna Briggs Institute (JBI) [19]. Two reviewers (CL and AH) independently conducted the quality assessment, with any discrepancies resolved through consensus.

The appropriate JBI checklist was applied according to the study design: randomized controlled trials, quasi-experimental studies, cohort studies, cross-sectional analyses, case series (with ≥5 participants), and qualitative research were all appraised using the corresponding JBI instruments. For mixed methods studies, both quantitative and qualitative components were assessed separately using the relevant JBI tools.

Each checklist evaluated key domains of methodological rigor, including selection bias, measurement validity, confounding, and clarity of outcome reporting. Studies were not excluded based solely on quality ratings; however, appraisal results were documented to guide the interpretation of the strength and credibility of the evidence. Findings from the quality assessments are presented in tabular format in Multimedia Appendix 2 and summarized narratively to highlight common methodological strengths and limitations across the included literature.

## Results

### Description of Studies

A total of 15,176 records were identified through electronic database searches. After removing 11,992 duplicates, 3184 unique titles and abstracts were screened for relevance. Of these, 2721 were excluded based on eligibility criteria during the initial title and abstract review. Seven additional full-text reports could not be retrieved, leaving 456 articles for full-text review.

During this stage, 445 reports were excluded: 12 due to an ineligible population, 371 for lacking a combined informatics and psychotherapeutic intervention, and 62 for using study designs outside the scope of the review.

**Figure 1.**
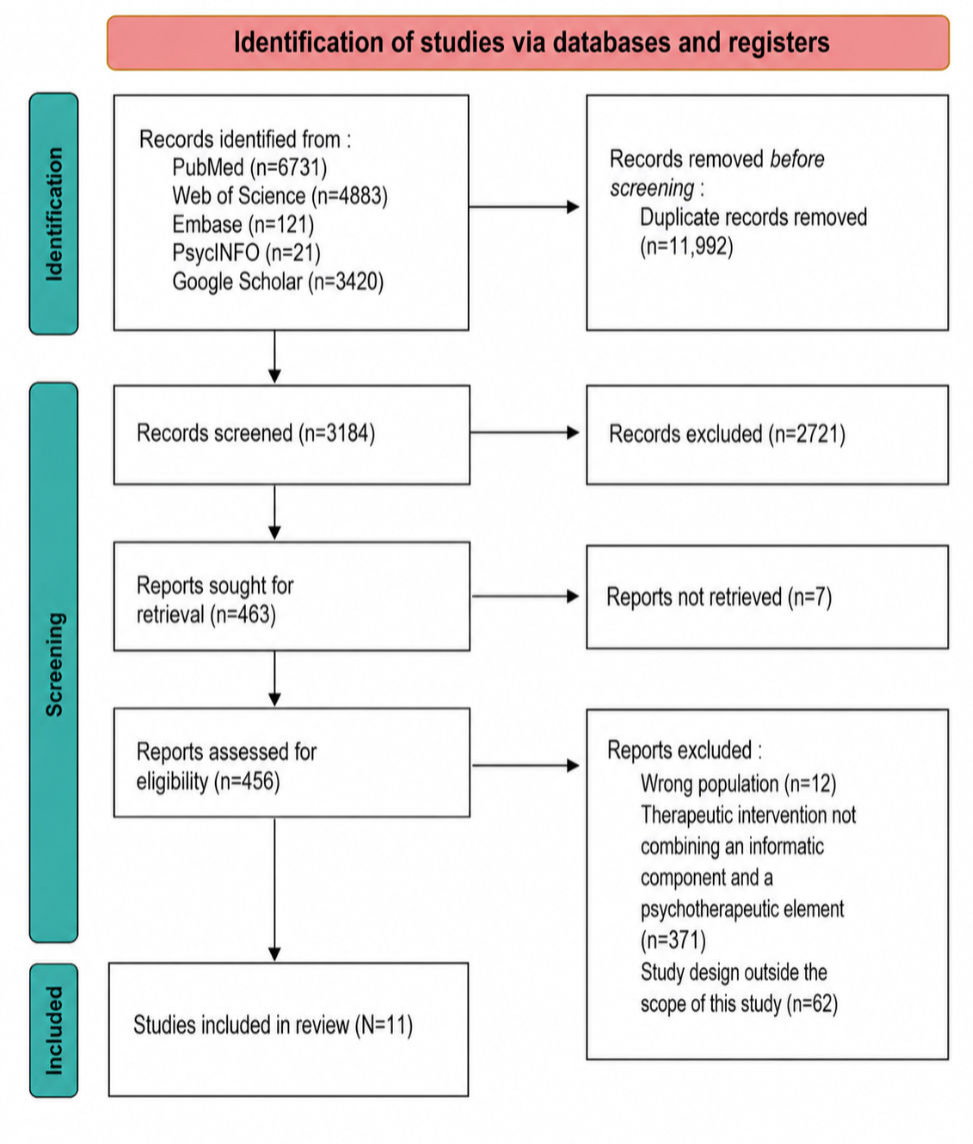
PRISMA (Preferred Reporting Items for Systematic Reviews and Meta-Analyses) flowchart illustrating the inclusion of studies.

Ultimately, 11 studies met all inclusion criteria and were included in the final synthesis. A detailed flowchart of the screening and selection process is presented in Figure 1, and key study characteristics are summarized in Multimedia Appendix 2.

### Main Outcomes

This scoping review synthesized findings from 11 peer-reviewed studies evaluating digital interventions with psychotherapeutic or psychiatric components aimed at improving both dermatologic and mental health outcomes. Most studies focused on chronic inflammatory skin diseases, especially psoriasis (n=9), with fewer addressing AD, HS, or vitiligo.

Digital platforms included iCBT, mindfulness-based self-help modules, mobile apps (eg, *DermaScope, Allay*), VR interfaces, and AI-assisted screening tools. Psychotherapeutic elements comprised guided iCBT, compassion-focused therapy, acceptance-based therapy, and self-guided mindfulness recordings, while psychiatric components involved consult-liaison psychiatry or psychotropic medication management within digitally integrated pathways.

Many studies reported statistically significant improvements in both dermatologic severity (eg, Psoriasis Area and Severity Index [PASI] scores, itch scales, DLQI) and mental health outcomes (eg, depression, anxiety, illness perceptions), though effect sizes and adherence rates varied. Dropout rates ranged from 10% to 46%, with generally high acceptability among users who completed the interventions. Most interventions were delivered as adjuncts to standard dermatologic care, but only a minority were integrated within structured care pathways.

Risk of bias was moderate in most studies, with common limitations including small sample sizes, nonblinded designs, and short follow-up periods. Despite these constraints, the evidence suggests that digital psychodermatologic interventions are both feasible and potentially beneficial, warranting further development and refinement. The main outcomes are summarized in Table 1.

**Table 1.** Main outcomes across the 11 identified peer-reviewed studies.

Study, year	Population with condition	Intervention summary	Key outcomes	Interaction modality
van Beugen et al, 2016 [20]	Adults with psoriasis	Tailored therapist-guided (iCBT^a^) targeting coping and disease adjustment	Improved physical functioning and daily life impact; mixed psychological effects	Hybrid (asynchronous + synchronous)
Hedman-Lagerlöf et al, 2021 [21]	Adults with AD^b^	Therapist-guided iCBT (12 wk)	Reduced AD severity, itch, stress, depression; sustained at 12 months	Hybrid (asynchronous + synchronous)
Kern et al, 2023 [22]	Adults with AD	Self-guided digital CBT^c^-based intervention (8 wk)	Improvements in QoL^d^, itch, depression, stress; feasibility demonstrated	Asynchronous
Kern et al, 2025 [12]	Adults with AD	Self-guided vs clinician-guided online CBT (RCT^e^)	Self-guided noninferior to clinician-guided; reduced symptoms (POEM^f^)	Hybrid (asynchronous + synchronous)
Fortune et al, 2022 [23]	Adults with psoriasis	Mobile app (*Allay*) delivering digital therapeutic content (CBT-informed)	Improved QoL, resilience, depression, illness perceptions	Asynchronous
Domogalla et al, 2021 [24]	Adults with psoriasis	Smartphone app + educational program integrated into care	Reduced depressive symptoms and QoL improvements (long-term)	Hybrid (asynchronous + synchronous)
Muftin et al, 2022 [25]	Adults with psoriasis	Online compassion-based and mindfulness-based self-help (RCT)	Reduced shame; improved QoL	Asynchronous
Clarke et al, 2024 [26]	Adults with skin conditions and depression	Online compassion-based guided self-help intervention	Improved depression, QoL, self-compassion; feasibility established	Hybrid (asynchronous + synchronous)
Sengupta and Wagani, 2025 [27]	Adults with various skin conditions	Online mindful self-compassion intervention (structured sessions)	Reduced depression, anxiety, stress; improved QoL and well-being	Synchronous (scheduled sessions)
Zhou et al, 2024 [28]	Adults with psoriasis	Online MBCT^g^ + usual care	Improved PASI^h^, depression, QoL, itch	Asynchronous
Leibovici et al, 2009 [29]	Adults with pruritus (AD/psoriasis)	VR^i^ immersion and audiovisual distraction	Reduced pruritus intensity (short-term)	Automated

a iCBT: internet-based cognitive behavioral therapy.

b AD: atopic dermatitis.

c CBT: cognitive behavioral therapy.

d QoL: quality of life.

e RCT: randomized controlled trial.

f POEM: Patient-Oriented Eczema Measure.

g MBCT: mindfulness-based cognitive therapy.

h PASI: Psoriasis Area and Severity Index.

i VR: virtual reality.

### Landscape of Digital Interventions

The digital interventions identified in this review encompassed a broad range of informatics modalities designed to support psychotherapeutic or psychiatric care in populations with dermatologic disorders.

Web-based iCBT was the most extensively studied approach. In the trial by van Beugen et al [20], a 12-week secure online platform delivered guided modules targeting stress, dysfunctional beliefs, and disease coping in patients with psoriasis, producing moderate improvements in quality of life and psychological flexibility compared to care-as-usual. Similarly, Fortune et al [23] embedded a therapist-supported iCBT program within the *Allay* mobile app, providing structured psychoeducation and skill-building for emotional distress in psoriasis, with early evidence of improved illness perceptions and emotional adjustment.

Compassion and mindfulness-based audio programs were examined in 2 studies. They were delivered via MP3 or digital links. Muftin et al [25] compared self-directed compassion-focused therapy with mindfulness-based therapy for psoriasis, with both interventions showing moderate reductions in distress and improved skin-related attitudes. Clarke et al [26] evaluated a guided audio intervention using kindness and acceptance-based recordings for patients with AD and HS, noting favorable user engagement and emotional improvements.

mHealth apps such as *DermaScope* and *Allay* were also common platforms [23,24]. *DermaScope* offered educational content and emotional support prompts, achieving high user satisfaction and reducing depressive symptoms in a German psoriasis cohort. *Allay*, implemented in the United States and Ireland, enabled daily symptom monitoring, psychoeducational content, and therapist communication, with most users requesting continued access after the study.

AI-driven tools were more exploratory. Zhou et al [28] developed a machine learning model to predict psychiatric comorbidity risk in HS, demonstrating the feasibility of AI-assisted mental health triage within dermatology.

Similarly, VR was used in Leibovici et al [29] as a distraction technique for patients experiencing severe itching, with immersive 3D environments reducing perceived discomfort during acute episodes.

Emerging modalities included Sengupta et al [27] conversational chatbot integrated with skin-tracking functions to provide CBT-based support to adolescents with vitiligo, which showed positive user attitudes and symptom control over time. Kern et al [12,22] piloted a novel e-psychiatry consult-liaison model for dermatology referrals, enabling synchronous psychiatric evaluation via teleplatforms, thereby improving continuity of care for unmet psychiatric needs.

### Target Populations and Diagnoses

Psoriasis was the primary focus in most included studies, with 9 out of 11 trials targeting individuals with moderate-to-severe plaque psoriasis. Participants were typically adults aged from 35 to 53 years, with a predominance of female respondents and relatively high levels of education. Several protocols assumed or prescreened for digital literacy, reflecting a bias toward digitally engaged populations. Few studies reported detailed breakdowns of socioeconomic status, race, or rural versus urban residence, limiting the generalizability to marginalized groups.

HS was the sole focus in Zhou et al [28], which applied AI-based stratification to improve psychiatric detection and triage. Sengupta et al [27] included a broader diagnostic mix, engaging adolescents and young adults with vitiligo, AD, and other pigmentary disorders via a chatbot-enabled support system. AD and chronic itch were secondarily mentioned in Clarke et al [26] and Leibovici et al [29], respectively, though without significant subgroup analyses.

No study targeted pediatric or geriatric patients exclusively, and few interventions were adapted for linguistic or cognitive accessibility. Clinical samples were largely restricted to mild-to-moderate symptom severity, and most trials excluded patients with active suicidality, substance use disorders, or psychosis. This suggests a significant gap in psychodermatologic research involving more complex or underserved patient populations.

### Outcomes

All included studies assessed at least 1 validated mental health outcome, most commonly using the Hospital Anxiety and Depression Scale, Patient Health Questionnaire-9, Brief Illness Perception Questionnaire, and visual analog scales for stress and emotional burden. Dermatologic outcomes were also consistently reported, with 8 studies using standardized measures such as the PASI, the Dermatology Life Quality Index (DLQI), and Numeric Rating Scale for itch and pain.

Several studies have demonstrated statistically significant, though modest, reductions in emotional distress. Domogalla et al [24] reported a mean 3.2-point decrease on the Patient Health Questionnaire-9 following a mobile app intervention, while van Beugen et al [20] found reduced self-reported helplessness and avoidance behavior using a digital CBT protocol. Fortune et al [23] observed improvements in illness beliefs (particularly in perceived control and emotional representation) following therapist-supported iCBT via the *Allay* app.

Multicomponent programs incorporating mindfulness or compassion elements yielded improvements not only in depression and anxiety scores but also in broader functional outcomes, such as sleep quality, social participation, and perceived stigmatization [25,26]. DLQI scores improved in 6 studies, with reductions of 3 to 7 points from baseline, indicating clinically meaningful gains in daily functioning and disease coping.

Long-term effects were less certain, as few studies included follow-up beyond 8 to 12 weeks. Notable exceptions included Hedman-Lagerlöf et al [21] who conducted a randomized controlled trial of exposure-based iCBT for AD, demonstrating sustained reductions in skin-related avoidance, anxiety, and depressive symptoms at a 12-month follow-up.

The study by Zhou et al [28] was the only one to focus on predicting psychiatric morbidity algorithmically, rather than evaluating therapeutic outcomes directly, underscoring a gap between predictive and interventional research.

Overall, while effect sizes varied, the evidence consistently pointed to positive impacts on both dermatologic and psychological well-being when digital tools incorporated structured therapeutic content. A summary of the findings is presented in Table 2.

**Table 2.** Summary of the findings.

Category	Summary
Most studied condition	Psoriasis (n=9 studies), with some inclusion of HS^a^, AD^b^, and vitiligo
Age range of participants	Adults aged 18‐65 y; mean range across studies: 35‐53 y
Gender representation	Majority female, digitally literate, higher education level
Digital modalities used	Web platforms, mobile apps, chatbots, VR^c^, AI^d^ triage tools, e-consults
Therapeutic approaches	CBT^e^, ACT^f^, mindfulness, compassion-based therapy, liaison psychiatry
Mental health outcomes measured	HADS^g^, PHQ-9^h^, B-IPQ^i^, stress and mood scales, QoL^j^ indicators
Dermatologic outcomes measured	PASI^k^, DLQI^l^, itch or pain scales, visual symptom tracking
Key mental health results	Small-to-moderate reductions in anxiety, depression, and distress; improved illness beliefs
Key dermatologic results	DLQI improved in 6 studies; itch and pain modestly reduced in short-term
Duration of interventions	From 10-min VR to 8- to 12-wk programs; mostly 4‐8 wk
Provider involvement	Ranged from self-guided applications to full psychiatric consults; minimal hybrid models
Barriers identified	Digital fatigue, limited literacy, emotional burden of disease reminders
Facilitators identified	Autonomy, ease of access, therapist support, personalization of content
Equity considerations	Minimal focus on older adults, underserved groups, or multilingual adaptation
Gaps in research	Lack of long-term outcomes, economic data, implementation strategies, equity, and digital alliance measures

a HS: hidradenitis suppurativa.

b AD: atopic dermatitis.

c VR: virtual reality.

d AI: artificial intelligence.

e CBT: cognitive behavioral therapy.

f ACT: acceptance and commitment therapy.

g HADS: Hospital Anxiety and Depression Scale.

h PHQ-9: Patient Health Questionnaire-9.

i B-IPQ: Brief Illness Perception Questionnaire.

j QoL: quality of life.

k PASI: Psoriasis Area and Severity Index.

l DLQI: Dermatology Life Quality Index.

### Implementation and Sustainability

The majority of interventions were designed as adjunctive supports rather than standalone treatments, aiming to complement rather than replace dermatologic care. Delivery was predominantly asynchronous, allowing users to access modules, audio content, or app-based features on their own schedules without real-time clinician involvement. Intervention duration varied widely—from a single 10-minute VR session for itch relief to 8- to 12-week therapist-supported iCBT or self-guided compassion-based therapy protocols [20,25,26,29]. Clinical involvement ranged from fully automated, self-guided platforms (eg, Sengupta 2025 chatbot, Domogalla mobile app) to integrated psychiatry consultations delivered via telehealth, as seen in Kern et al [12], where dermatologists referred patients to e-psychiatry services for diagnostic clarification and psychotropic management [22].

Reported barriers to implementation included digital fatigue (with daily app reminders or disease-focused content perceived as burdensome) [23,25], technological literacy challenges, and data privacy concerns, particularly among older or digitally underserved populations [28]. Dropout rates ranged from 10% to 46%, with reasons for attrition often unreported or multifactorial. Conversely, facilitators of engagement included content personalization, 24/7 accessibility, and a sense of autonomy and empowerment, especially in self-help platforms [24,26].

Despite these insights, few studies examined long-term sustainability, cost-effectiveness, or integration into routine dermatology workflows. Only Kern et al [12,22] assessed provider workflow impact, and no study conducted formal equity analyses by race, income, rurality, or language. Overall, while the evidence supports feasibility, substantial gaps remain in implementation science and health equity evaluation for digital psychodermatology.

### Guidance Level and Adherence

Across the included studies, interventions varied substantially in the degree of human support provided, ranging from fully self-guided (unguided) digital interventions to clinician- or therapist-supported (guided) models, with several hybrid approaches combining asynchronous content with limited human interaction. Guided interventions, including therapist-supported iCBT and structured clinician feedback embedded within digital platforms, were reported in studies such as Hedman-Lagerlöf et al [21] and van Beugen et al [20], where participants received periodic support, feedback, or monitoring alongside digital modules. In contrast, unguided interventions—including self-help mobile apps and fully automated online programs (eg, Fortune et al [23], Muftin et al [25], and Kern et al [12] self-guided arm)—relied primarily on user-driven engagement without real-time or personalized clinician input [20,21]. Hybrid models, such as those incorporating minimal email support or periodic check-ins (eg, [24,26]), occupied an intermediate position in terms of resource intensity. When examined in relation to adherence, a pattern emerged whereby studies reporting higher levels of human support tended to demonstrate lower attrition and more sustained engagement, whereas fully unguided interventions were more frequently associated with higher dropout rates [24,26]. For example, therapist-guided iCBT trials reported relatively stable completion rates despite minimal clinician time investment, while several self-guided interventions noted usability challenges or attrition over time. Overall dropout rates varied across studies with variability partly attributable to differences in intervention intensity, duration, and user support. Although the heterogeneity of study designs and reporting precludes formal quantitative comparison, these findings suggest a potential association between the degree of human support and intervention adherence, with guided or hybrid models appearing to mitigate disengagement relative to fully self-directed approaches.

### Quality Appraisal

Using JBI appraisal tools, most studies demonstrated a moderate risk of bias. Randomized controlled designs were generally of higher methodological quality but remained susceptible to open-label effects and attrition [20,25]. Quasi-experimental and observational studies provided useful insights but were limited by small sample sizes and short follow-up periods [24,27]. Overall, quality ratings across studies ranged from moderate to high based on the JBI checklists. Detailed assessments are presented in Multimedia Appendix 2.

### Knowledge Gaps

A number of critical knowledge gaps were identified across the reviewed literature. First, the demographic reach of interventions was limited: most samples comprised middle-aged, digitally literate adults, with minimal inclusion of older adults, individuals with low health or digital literacy, or patients from rural or socioeconomically disadvantaged backgrounds. No study provided stratified analyses by race, ethnicity, or income, and none were explicitly designed to address the needs of underserved populations.

While several interventions demonstrated short-term clinical or psychological benefits, none assessed long-term psychiatric outcomes, such as suicidal ideation, substance use disorders, or psychiatric hospitalization, despite the elevated prevalence of these conditions in chronic dermatologic illnesses. Furthermore, sustainability and cost-effectiveness were rarely addressed. Only Kern et al [12] investigated system-level integration into dermatologic workflows, and no study conducted a formal economic evaluation or budget impact analysis.

There was also a notable absence of key implementation metrics, such as intervention fidelity, training burden, and reimbursement feasibility, limiting the applicability of findings to real-world clinical settings. Although several interventions were delivered via mobile apps or web-based portals, blended care models that combine digital and in-person therapeutic components remain largely unexplored.

Additionally, the digital therapeutic alliance, a key factor in mental health treatment efficacy, was not systematically assessed in any study. Equity-focused implementation strategies, including culturally adapted content or multilingual delivery formats, were entirely absent.

Addressing these gaps is essential to ensure that psychodermatological digital tools are scalable, inclusive, and clinically meaningful across diverse patient populations and care systems.

## Discussion

### Principal Results

This scoping review synthesized findings from 11 studies evaluating informatics-enabled psychotherapeutic or psychiatric interventions in dermatology. Most interventions were delivered via digital platforms, including web-based cognitive CBT, mHealth apps, mindfulness audio modules, VR tools, and AI-supported systems. The observed effects of digital psychodermatologic interventions can be interpreted through the lens of the brain-skin axis, a bidirectional neuroimmunological pathway linking psychological stress and cutaneous inflammation. By reducing stress and improving coping and behavioral responses (eg, scratching), interventions such as iCBT may attenuate activation of the hypothalamic-pituitary-adrenal axis and downstream inflammatory processes, contributing to improvements in both mental health and skin severity (eg, PASI). This mechanistic framework supports the integration of digital mental health tools as adjunctive strategies in the management of chronic inflammatory skin diseases.

Psoriasis emerged as the most frequently studied dermatologic condition, while fewer studies addressed AD, HS, or mixed skin disorders. All studies assessed mental health outcomes, most commonly targeting depression, anxiety, stress, or illness perception. Several interventions demonstrated moderate improvements in emotional distress, self-efficacy, and dermatologic quality of life, particularly those integrating CBT or self-compassion frameworks.

However, digital equity considerations, long-term adherence, and integration into routine dermatologic care were rarely assessed. Methodological quality was generally moderate to high, but many studies were underpowered, lacked control groups, or experienced high attrition.

### Comparison With Prior Work

Psychological therapies have demonstrated measurable benefits in chronic skin conditions such as psoriasis [30]. A recent systematic review of randomized trials found that interventions such as cognitive behavioral therapy (CBT), mindfulness-based interventions, and structured patient education can improve both mental health outcomes and dermatologic severity in psoriasis [31]. Similarly, Qureshi et al [30] reported that adjunctive psychological programs (including stress management and biofeedback) were associated with better skin clearance and quality-of-life improvements in psoriatic disease. However, most interventions in these studies were delivered in person, with only a minority using digital or telehealth formats [31]. This gap highlights an underexplored opportunity in dermatology, given the logistical advantages of digital platforms for chronic care.

Our review highlights the unique value of informatics platforms (such as web portals and mobile apps) in psychodermatology. Digital tools can reduce access barriers and extend care beyond clinic visits through on-demand support and self-management resources. Unlike weekly face-to-face sessions, online programs can provide asynchronous, low-cost coaching that patients can access between dermatology appointments. Evidence from general mental health research suggests this convenience does not necessarily compromise efficacy: multiple meta-analyses have shown that guided iCBT produces outcomes equivalent to those of in-person therapy for anxiety and depression [32]. Andersson and colleagues [32] further demonstrated that across conditions, iCBT achieves symptom reductions comparable to face-to-face care, although direct comparisons remain limited.

Despite these findings, dermatology still lacks head-to-head trials explicitly comparing digital and traditional psychotherapeutic delivery. This research underscores the importance of formally evaluating whether internet-delivered psychotherapies can match the clinical outcomes of office-based care in populations with dermatologic disorders.

Digital CBT has also proven effective in other chronic medical conditions, such as improving depression in diabetes and reducing pain catastrophizing in arthritis [33]. However, adaptation of these interventions to dermatology-specific needs remains limited. Content must be tailored to address skin-related stressors, such as visible lesions, itch-scratch cycles, and social stigma. Yet, personalized psychodermatology programs remain rare, with few adapted to specific clinical phenotypes (eg, itch-dominant psoriasis or high-stigma AD), which reinforces the call for more personalized digital therapeutics. As Kazdin [34] has argued in his framework for mental health innovation, digital platforms offer a unique opportunity to personalize therapy content to individual patient profiles at scale, which is an important direction for future research.

A striking finding of this review is the disproportionate focus on psoriasis, which accounted for 9 of the 11 included studies, with comparatively minimal representation of other high-burden dermatologic conditions such as AD and HS. This imbalance likely reflects several structural drivers within the field. First, psoriasis benefits from a long-standing research infrastructure, including the widespread use of standardized and validated outcome measures such as the PASI and the DLQI, which facilitate trial design, comparability, and regulatory alignment. In contrast, while AD has measures such as the Patient-Oriented Eczema Measure and Eczema Area and Severity Index, and HS has emerging indices such as Hidradenitis Suppurativa Clinical Response, these tools have been less consistently integrated into psychodermatology and digital intervention research, potentially limiting study development and cross-study synthesis. Second, psoriasis has been a primary target of substantial pharmaceutical investment, particularly with the expansion of the biologics market, which has historically driven broader innovation ecosystems, including adjunctive behavioral and digital interventions. This contrasts with conditions such as HS, which, despite significant psychosocial burden and high rates of psychiatric comorbidity, has received comparatively less research funding and fewer large-scale intervention studies. Third, psoriasis populations are often easier to recruit within structured clinical pathways and registries, enabling digital trial implementation, whereas patients with other conditions may be more fragmented across care settings. The consequence is a literature that may overrepresent conditions with strong biomedical and commercial ecosystems while underrepresenting those with equally or greater psychosocial need. This raises important concerns regarding the generalizability of current findings and highlights a critical gap in the development of digital psychodermatologic interventions for underserved patient populations. Future research should prioritize conditions such as HS and AD not only to ensure equity in innovation but also to better align digital intervention development with the true distribution of psychological burden across dermatologic diseases.

The present findings should be interpreted in light of the mixed methods systematic review by Hewitt et al [35], which represents the most closely related synthesis of digital psychological interventions in dermatology and identified 23 studies, including 15 randomized controlled trials, concluding that such interventions may improve psychological outcomes such as mood, quality of life, and illness-related knowledge despite substantial heterogeneity limiting definitive conclusions. While both reviews examine the intersection of digital technologies and psychological support in populations with dermatologic disorders, this study extends and refines this evidence base through several key distinctions. Our inclusion criteria were deliberately more restrictive, requiring interventions to integrate both an informatics component and a structured psychotherapeutic or psychiatric element, and to report dermatologic and/or mental health outcomes; this approach excludes purely educational or communication-based tools without explicit therapeutic intent, thereby enhancing clinical interpretability despite yielding a smaller number of included studies (n=11). The review also extends the literature temporally and conceptually by incorporating studies published up to March 2025, capturing the emergence of newer modalities such as AI-assisted tools, virtual assistants, and hybrid care models that were largely absent from earlier syntheses and signal a shift toward more interactive, personalized, and scalable psychodermatologic interventions. In contrast to prior work, this synthesis explicitly treats dermatologic and psychological outcomes as coprimary domains, integrating clinical severity indices (eg, PASI, DLQI) with validated mental health measures to adopt a more comprehensive biopsychosocial perspective that reflects the bidirectional relationship between skin disease and psychological distress. Additionally, greater emphasis is placed on implementation considerations, including adherence, integration into clinical workflows, and equity-related barriers; although prior work identified issues of acceptability and usability, this analysis further examines how such interventions are operationalized in practice and highlights persistent gaps, particularly regarding long-term engagement and access among underserved populations. Taken together, this review does not contradict prior findings but rather refines and extends them through a more clinically focused definition of intervention, the inclusion of emerging technologies, and the positioning of outcomes within an integrated dermatologic-psychiatric framework, thereby contributing to the advancement of scalable and clinically embedded models of psychodermatologic care.

### Limitations

This scoping review has several limitations. Despite a comprehensive multidatabase search strategy, relevant studies may have been missed, particularly those published in nonindexed journals, in languages other than English or French, or under alternative terminologies not captured by our search terms.

The heterogeneity in study designs, populations, interventions, and outcomes limited our ability to perform quantitative synthesis or direct cross-study comparisons. Many included studies had small sample sizes, high attrition rates, and limited follow-up, reducing the generalizability and robustness of their findings. Most trials also lacked standardized measures of adherence, usability, or cost-effectiveness, which are essential for assessing scalability. Also, a key finding of this review is the widespread lack of reporting on sociodemographic variables such as race or ethnicity, education, and socioeconomic status, despite frequent references to barriers like digital literacy. This gap limits the interpretability and generalizability of findings and raises concerns that current evidence may disproportionately reflect more digitally literate and advantaged populations, underscoring the need for standardized reporting and more inclusive study designs.

This review did not systematically include gray literature, which may have excluded unpublished or program-level innovations from clinical settings. Finally, although we aimed to capture both dermatological and mental health outcomes, few studies reported comprehensively on both, and even fewer examined long-term effects or implementation into routine dermatological care.

### Conclusions

This scoping review provides a comprehensive synthesis of the emerging field of informatics-based psychotherapeutic and psychiatric interventions in dermatology. The reviewed studies highlight the feasibility and preliminary efficacy of digital tools in addressing both psychological distress and dermatologic symptom burden across a range of chronic skin conditions. These interventions offer accessible and scalable, patient-centered models of care that can complement traditional dermatology services, particularly in resource-limited or remote settings.

However, this evidence base is constrained by methodological heterogeneity, small sample sizes, high attrition, and insufficient reporting on equity, usability, and long-term impact. The lack of integration into dermatologic workflows and the absence of head-to-head comparative studies with face-to-face psychotherapy also represent additional knowledge gaps.

Future research should prioritize designing trials that evaluate digital interventions in diverse populations, incorporate equity-informed frameworks, and test hybrid models of care that blend digital tools with human support. As the fields of psychodermatology and digital health continue to converge, the development of personalized, adaptable, and clinically integrated digital mental health interventions will be essential to improving patient outcomes and reducing disparities in dermatologic care.

## Supplementary material

10.2196/82096 Multimedia Appendix 1 Electronic search strategy for the scoping review conducted.

10.2196/82096 Multimedia Appendix 2 Systematic review study selection detailed results.

10.2196/82096 Checklist 1 PRISMA-ScR checklist.

## Abbreviations

AD: atopic dermatitis
AI: artificial intelligence
CBT: cognitive behavioral therapy
DLQI: Dermatology Life Quality Index
HS: hidradenitis suppurativa
iCBT: internet-based cognitive behavioral therapy
JBI: Joanna Briggs Institute
PASI: Psoriasis Area and Severity Index
PRISMA-ScR: Preferred Reporting Items for Systematic Reviews and Meta-Analyses extension for Scoping Reviews
VR: virtual reality
mHealth: mobile health
