# Supplementary material for: Informatics-Based Psychotherapeutic and Psychiatric Interventions in Dermatology: Scoping Review of Impacts on Skin Disease Severity and Mental Health Outcomes
Source: JMIR Dermatol. 2026 Jun 10;9:e82096. doi: 10.2196/82096 (PMC13252885; doi:10.2196/82096)
Supplement: Multimedia Appendix 1 [file derma-v9-e82096-s001.docx]

**Supplementary Online Content**

Lamarre, C., Chivinski, J, Hudon, A. Informatics-Based Psychotherapeutic and Psychiatric Interventions in Dermatology: A Scoping Review of Impacts on Skin Disease Severity and Mental Health Outcomes

**Multimedia Appendix 1.** Electronic search strategy for the scoping review conducted.

**Multimedia Appendix 1. Electronic search strategy for the scoping review conducted.**

| **Database; Search** | **Search Terms** |
| --- | --- |
|  |  |
| PubMed; k= 6731 | (("psoriasis"[Title/Abstract] OR "atopic dermatitis"[Title/Abstract] OR "eczema"[Title/Abstract] OR "hidradenitis suppurativa"[Title/Abstract] OR "acne"[Title/Abstract] OR "vitiligo"[Title/Abstract] OR "alopecia"[Title/Abstract] OR "chronic urticaria"[Title/Abstract])  AND  ("digital health"[Title/Abstract] OR "teledermatology"[Title/Abstract] OR "mobile application"[Title/Abstract] OR "mHealth"[Title/Abstract] OR "eHealth"[Title/Abstract] OR "internet-based therapy"[Title/Abstract] OR "internet-based CBT"[Title/Abstract] OR "iCBT"[Title/Abstract] OR "digital therapeutics"[Title/Abstract] OR "virtual care"[Title/Abstract] OR "chatbot"[Title/Abstract] OR "AI"[Title/Abstract] OR "conversational agent"[Title/Abstract] OR "psychiatry e-consult"[Title/Abstract])  AND  ("psychotherapy"[Title/Abstract] OR "mental health"[Title/Abstract] OR "psychological outcomes"[Title/Abstract] OR "depression"[Title/Abstract] OR "anxiety"[Title/Abstract] OR "stress"[Title/Abstract] OR "quality of life"[Title/Abstract] OR "CBT"[Title/Abstract] OR "mindfulness"[Title/Abstract] OR "psychoeducation"[Title/Abstract] OR "consult-liaison psychiatry"[Title/Abstract])) |
| Web of Science; k=4883 | TS=("psoriasis" OR "atopic dermatitis" OR "eczema" OR "hidradenitis suppurativa" OR "acne" OR "vitiligo" OR "alopecia" OR "chronic urticaria")  AND  TS=("digital health" OR "teledermatology" OR "mobile application" OR "mHealth" OR "eHealth" OR "internet-based therapy" OR "internet-based CBT" OR "iCBT" OR "web-based intervention" OR "digital therapeutics" OR "virtual care" OR "chatbot" OR "AI-supported" OR "artificial intelligence" OR "conversational agent" OR "psychiatry e-consult")  AND  TS=("psychotherapy" OR "mental health" OR "depression" OR "anxiety" OR "stress" OR "quality of life" OR "CBT" OR "mindfulness" OR "psychoeducation" OR "consult-liaison psychiatry" OR "psychological outcomes") |
| Embase; k = 121 | ('psoriasis':ti,ab OR 'atopic dermatitis':ti,ab OR 'eczema':ti,ab OR 'hidradenitis suppurativa':ti,ab OR 'acne':ti,ab OR 'vitiligo':ti,ab OR 'alopecia':ti,ab OR 'chronic urticaria':ti,ab)  AND  ('digital health':ti,ab OR 'teledermatology':ti,ab OR 'mobile application':ti,ab OR 'mhealth':ti,ab OR 'ehealth':ti,ab OR 'internet-based therapy':ti,ab OR 'internet-based cbt':ti,ab OR 'icbt':ti,ab OR 'digital therapeutics':ti,ab OR 'virtual care':ti,ab OR 'chatbot':ti,ab OR 'ai':ti,ab OR 'conversational agent':ti,ab OR 'psychiatry e-consult':ti,ab)  AND  ('psychotherapy':ti,ab OR 'mental health':ti,ab OR 'psychological outcomes':ti,ab OR 'depression':ti,ab OR 'anxiety':ti,ab OR 'stress':ti,ab OR 'quality of life':ti,ab OR 'cbt':ti,ab OR 'mindfulness':ti,ab OR 'psychoeducation':ti,ab OR 'consult-liaison psychiatry':ti,ab) |
| PsycINFO; k = 21 | ((psoriasis OR "atopic dermatitis" OR eczema OR "hidradenitis suppurativa" OR acne OR vitiligo OR alopecia OR "chronic urticaria")  AND  ("digital health" OR teledermatology OR "mobile application" OR mHealth OR eHealth OR "internet-based therapy" OR "online therapy" OR "internet-based CBT" OR iCBT OR "digital therapeutics" OR "virtual care" OR chatbot OR AI OR "artificial intelligence" OR "conversational agent" OR "psychiatry e-consult")  AND  (psychotherapy OR "mental health" OR depression OR anxiety OR stress OR "quality of life" OR CBT OR mindfulness OR psychoeducation OR "consult-liaison psychiatry")) |
| Google Scholar; k= 3420 | ("psoriasis" OR "atopic dermatitis" OR "hidradenitis suppurativa" OR "dermatology" OR "skin condition" OR "skin disorder")  AND  ("internet-based CBT" OR iCBT OR "teledermatology" OR "digital therapy")  AND  ("mental health" OR "psychotherapy") |
