# Supplementary material for: Informatics-Based Psychotherapeutic and Psychiatric Interventions in Dermatology: Scoping Review of Impacts on Skin Disease Severity and Mental Health Outcomes
Source: JMIR Dermatol. 2026 Jun 10;9:e82096. doi: 10.2196/82096 (PMC13252885; doi:10.2196/82096)
Supplement: Multimedia Appendix 2 [file derma-v9-e82096-s002.docx]

**Supplementary Online Content**

Lamarre, C., Chivinski, J, Hudon, A. Informatics-Based Psychotherapeutic and Psychiatric Interventions in Dermatology: A Scoping Review of Impacts on Skin Disease Severity and Mental Health Outcomes

**Multimedia Appendix 2.** Scoping review study selection detailed results.

This supplementary material has been provided by the authors to give readers additional information about their work.

**Table S2. Systematic review study demographics detailed results.**

| **Authors** | **Country** | **Study Design** | **Sample Size** | **Dermatologic Diagnosis** | **Baseline Severity** | **Mean Age of Participants** | **Age Range** | **Sex Distribution** | **Ethnicity** |
| --- | --- | --- | --- | --- | --- | --- | --- | --- | --- |
| van Beugen et al., 2016 [20] | Netherlands | Multicenter RCT | 131 (65 ICBT + CAU, 66 CAU) | Psoriasis | Mean PASI 5.99 (ICBT) / 4.20 (CAU) - mild-moderate | 53 | 19-79 | 51% male (67/131) | NR |
| Domogalla et al., 2021 [24] | Germany | Intervention study | 107 (54 IG + 53 CG) | Psoriasis | Mean HADS-D 5.3 (IG)/ 5.2 (CG), Mean HADS-A 6.7 (IG)/ 7.0 (CG), Mean DLQI 7.9 (IG)/ 8.5 (CG), Mean PASI 5.0 (IG)/ 5.1 (CG) | 49.1 | 36-59 | 61% male (65/107) | NR |
| Fortune et al., 2022 [22] | Ireland | Multicenter PoC | 66 | Psoriasis | Mean B-IPQ life impact 6.10, Mean DLQI 0.78, Mean BRS 3.06, Mean MHC-SF 48.19, Mean PHQ-4A 0.39, Mean PHQ-4D 0.29 | US 40.0, Ireland 38.9 | 18-64 | 50% male (33/32/1) | NR |
| Leibovici et al., 2009 [26] | Israel | Randomized Cohort study | 24 | Psoriasis | Score <5/10 VAS itching | 44.5 | 18-84 | 50% male (12/12) | NR |
| Muftin et al., 2022 [22] | England | Randomized Cohort study | 130 (65 compassion + 65 mindfulness | Psoriasis | Self-reported psoriasis severity scale; 79% of sample reported 5-9/9 severity | NR | 16-66 | 66% female (87/43 | 85% white, 15% Black/asian/mixed |
| Zhou et al., 2024 [25] | China | Single-site RCT | 109 (TAU 56, TAU + MBCT 53 | Psoriasis | Early-onset psoriasis | 34 | 19-69 | 50.6% female | NR |
| Hedman-Lagerlöf et al., 2021 [2021] | Sweden | Single-site RCT | 102 (CAU, CAU+CBT) | Atopic dermatitis | Moderate-severe AD | 37 | 18-59 | 83% female (81/19) | NR |
| Kern et al., 2023 [28] | Sweden | Interventional study | 21 | Atopic dermatitis | Moderate-severe AD | 42 | 21-62 | 95% female (20/21) | NR |
| Kern et al., 2025 [12] | Sweden | Single-site blind Randomized trial | 168 (86 SGG, 82 CGG) | Atopic dermatitis | Moderate-severe AD | 39 | 18-65 | 85% female (142/26) | NR |
| Clarke et al., 2024 [23] | England | Interventional study | 34 | Skin conditions | Skin conditions with mild-moderate depressive symptoms | 38 | 21-61 | 91% female | 82% white |
| Sengupta et al, 2025 [27] | India | Multicenter RCT | 208 | Skin conditions | Skin condition for minimally 6 months | NR | 18-55 | 67% female (59/29) | NR |

*Acronyms: AD: Atopic Dermatitis; B-IPQ: Brief Illness Perception Questionnaire; BRS: Brief Resilience Scale; CAU: Care As Usual; CBT: Cognitive Behavioral Therapy; CG: Control Group; CGG: Compassion Group Guided; DLQI: Dermatology Life Quality Index; HADS-A: Hospital Anxiety and Depression Scale - Anxiety subscale; HADS-D: Hospital Anxiety and Depression Scale - Depression subscale; ICBT: Internet-Based Cognitive Behavioral Therapy; IG: Intervention Group; MBCT: Mindfulness-Based Cognitive Therapy; MHC-SF: Mental Health Continuum - Short Form; NR: Not Reported; PASI: Psoriasis Area and Severity Index; PHQ-4A: Patient Health Questionnaire - Anxiety subscale; PHQ-4D: Patient Health Questionnaire - Depression subscale; PoC: Proof of Concept; RCT: Randomized Controlled Trial; SGG: Self-Guided Group; TAU: Treatment As Usual; VAS: Visual Analogue Scale.*

**Table S4. Systematic review study designs detailed results.**

| **Authors** | **Comorbid Mental Health** | **Recruitment Setting** | **Informatics Modality** | **Psychiatric Component Type** | **Delivery Mode** | **Dose Duration** | **Comparator** | **Theoretical Framework** |
| --- | --- | --- | --- | --- | --- | --- | --- | --- |
| van Beugen et al., 2016 [20] | Elevated distress (ISDL); DSM comorbidity excluded | Outpatient dermatology departments (academic & non-academic hospitals) + Dutch Psoriasis Association | Secure web-based platform (Internet-based CBT) | Individually tailored CBT modules | Therapist-guided asynchronous; initial 2 face-to-face sessions; weekly written feedback | Mean 25±12 weeks (range 1-57) | Care as usual | Cognitive Behavioral Therapy |
| Domogalla et al., 2021 [24] | Mood, daily activity, alcohol; excluding inability to provide written informed consent and no access to a smartphone | Outpatient clinic at the Department of Dermatology, Venereology, and Allergology at the University Medical Center Mannheim, Germany, between January 2018 and June 2020. | Psoriasis monitoring smartphone app DermaScope Mobile | | 2-hour-long educational program on the topic of psoriasis, which was held by specialists in dermatology; patients could contact specialized dermatologists unrestrictedly via a chat feature within the app. | 60 weeks | Care as usual | NR |
| Fortune et al., 2022 [22] | Anxiety, depression; Excluding no literacy, cognitive impairment, active CBT-like therapy, drugs misuse, history or current suicidal ideation | Outpatient dermatology departments (Ireland and New York departments) | Allay m-health intervention mobile app | CBT + IBSR activities and chat-bot support | App activation and induction measures, then, Allay digital therapeutic intervention | 12 weeks | Baseline | CBT + IBSR |
| Leibovici et al., 2009 [26] | NR | Outpatient (18) + hospitalized (6) dermatology departments (Jerusalem, Israel) | Interactive game AVD vs VRI | Distraction | 8-12 minutes depending on person's ability to complete game | NR | VAS itching + interviewer rate scratching on a 3 points scale | VRI + AVD |
| Muftin et al., 2022 [22] | Excluded if engaged in psychological therapy <=6M | Web-based psoriasis organization and University of Sheffield mailing list | MP3-file recorded modules | Feeling of shame | Compassion-based self-help vs mindfulness-based self-help | 4 weeks | Mindfulness vs self-compassion | Mindfulness + CMT |
| Zhou et al., 2024 [25] | Anxiety, depression; excluded severe mental illness and current psychotic drug use | outpatient dermatology department | weekly 60 mins. audio session recorded by psychiatrist | Anxiety, depression | Weekly 60 MCBT audio session, feedback, motivation and technical support | 8 weeks | Care as usual | MBCT |
| Hedman-Lagerlöf et al., 2021 [2021] | Include patients on stable doses antidepressant or sleep medication >1M; exclude cancer, severe psychiatric treatment, benzodiazepine use, ongoing/recent psychological treatment, psoriasis. | outpatient dermatology department medical university (Stockholm, Sweden) | secure and encrypted internet-based treatment platform | Anxiety, depression, stress, insomnia | Therapist-guided internet-delivery CBT, 10 modules, asynchronous feedback and support | 12 weeks | Care as usual | CBT |
| Kern et al., 2023 [28] | NR | telephone to nation wide AD patients + social-media advertisement | online homework assignment | Stress, depression | Self-management digital CBT-based intervention with telephone calls before and after | 8 weeks | Previously published study | CBT |
| Kern et al., 2025 [12] | NR | telephone to nation wide AD patients + social-media advertisement | online self-guided CBT program | Insomnia, stress | 10 modules, education material and homework | 12 weeks | Clinical-guided intervention | CBT |
| Clarke et al., 2024 [23] | Mild-moderate despression symptoms; excluding severe psychological illness and alcohol-drug use | online via social media and advertisement in skin condition organisations | Website and email support | Depression, self-compassion | Weekly compassion-based online intervention and email support | 6 weeks | Care as usual | CFT |
| Sengupta et al, 2025 [27] | Include psychological distressl exclude suicide ideas, psychological treatment, mindfulness meditation | 2 Mumbai private dermatology clinics | Email enrollment and website interventions/questionnaire | Depression, self-esteem | Bi-weekly 50 mins mindful self-compassion intervention modules | 4 weeks | Care as usual | Mindfulness |

*Acronyms: AD: Atopic Dermatitis; AVD: Alternative Video Distraction; AVR: Active Video Relaxation; CBT: Cognitive Behavioral Therapy; CFT: Compassion-Focused Therapy; CMT: Compassionate Mind Training; DSM: Diagnostic and Statistical Manual of Mental Disorders; IBSR: Inquiry-Based Stress Reduction; ISDL: Inventory of Skin-Related Distress in Daily Life; MBCT: Mindfulness-Based Cognitive Therapy; MHC-SF: Mental Health Continuum - Short Form; NR: Not Reported; VAS: Visual Analogue Scale; VRI: Virtual Reality Intervention.*

**Table S5. Systematic review study outcomes detailed results.**

| **Authors** | **Dermatologic Outcomes** | **Mental Health Outcomes** | **Adherence Metrics** | **Effect Estimates** | **Follow-Up Duration** | **Delivery Context** | **Technology platform used** |
| --- | --- | --- | --- | --- | --- | --- | --- |
| van Beugen et al., 2016 [20] | Physical functioning composite; fatigue; itch; PASI; SAPASI | Psychological functioning composite; negative mood; anxiety; BDI | Dropout 26% (15% non-starters; 9% during); mean satisfaction 7.6/10 | ICBT vs CAU: physical functioning p=0.03 d=0.36; impact on daily activities p=0.04 d=0.35 | 6 months | Adjunct to standard dermatologic care at hospitals | Secure intervention website; personal login |
| Domogalla et al., 2021 [24] | DLQI, PASI, Pain, pruritus | HADS-D, HADS-A, Mood, daily activity, app use frequency | Dropout 28% (0% non-starters; 28% during) |  | NR | Adjunct to standard dermatologic care at hospitals | personal anonymized access code DermaScope app |
| Fortune et al., 2022 [22] | B-IPQ, DLQI | BRS, MCH-SF, PHQ-4A, PHQ-4D | Dropout 46% (11% non-starters; 38% during) | Allay vs baseline: emotional impact p=0.005 d=0.62; impact of psoriasis p=0.001 d=.73; resilience level p=0.01 d=0.45; Sx of depression p=0.005 d=0.66; sx of psoriasis p=0.01 d= 45; QoL p=0.003 d=1.80 | NR | Adjunct to standard dermatologic care at hospitals | Amalgam Rx USA's *Allay* app |
| Leibovici et al., 2009 [26] | Pruritus | Distraction/immersion | Dropout 10% (3 non-starters) |  | NR | Adjunct to standard dermatologic care at hospitals | VRI eMagin Z8003DVisor; AVD computer and it's speakers; commercial interactive computer game with 10 levels |
| Muftin et al., 2022 [22] | DLQI | Feeling of shame + OAS ; FSCRS; Cronbach's alpha value | Dropout 30% (29% non-starters; 1% during) |  | NR | 10-15 minutes per self-help technique recorded information material | email with assigned MP3-files recorded techniques |
| Zhou et al., 2024 [25] | PASI, DLQI, pruritus | SAS, SDS | Dropout 17% follow up |  | 12 weeks | Adjunct to standard dermatologic care at hospitals | email |
| Hedman-Lagerlöf et al., 2021 [2021] | POEM, itching VAS, DLQI | PSS, PHQ-9, ISI, BAI, BBQ | Dropout 17% posttreatment follow-up | CAU+ CBT: Reduction of symptoms p=0.01; controlled effect after treatment d=0.75 | 1 year | Adjunct to standard dermatologic care at hospitals | email access to encrypted internet platform |
| Kern et al., 2023 [28] | POEM, PPNRS, DLQI | PSS, PHQ-9 | Dropout 14% posttreatment, 5% follow-up |  | 12 weeks | Adjunct to standard dermatologic care at hospitals | secure website |
| Kern et al., 2025 [12] | POEM, PPNRS, DLQI | ISI, PSS, PHQ-9, | Dropout 10% |  | 12 weeks | Adjunct to standard dermatologic care at hospitals | website |
| Clarke et al., 2024 [23] | DLQI | DASS-D, SCS | Dropout 76% (26% non-starters, 50% follow-up) | Increased SCS p=0.15; decreased DLQI p=0.04; decreased DASS-D p=0.15 | NR | Adjunct to standard dermatologic care at hospitals | website |
| Sengupta et al, 2025 [27] | DLQI | DASS-21, Self-esteem scale | Dropout 58% | Decreased levels of anxiety-depression-stress and increased levels of self-esteem/well-being/DLQI p=<0.001 | NR | Adjunct to standard dermatologic care at hospitals | website |

*Acronyms: AVD: Alternative Video Distraction; BAI: Beck Anxiety Inventory; BBQ: Brunnsviken Brief Quality of Life Scale; BDI: Beck Depression Inventory; B-IPQ: Brief Illness Perception Questionnaire; BRS: Brief Resilience Scale; CAU: Care as Usual; DASS-D: Depression Anxiety Stress Scale - Depression subscale; DLQI: Dermatology Life Quality Index; FSCRS: Forms of Self-Criticizing/Attacking and Self-Reassuring Scale; HADS-A: Hospital Anxiety and Depression Scale - Anxiety; HADS-D: Hospital Anxiety and Depression Scale - Depression; ICBT: Internet-based Cognitive Behavioral Therapy; ISI: Insomnia Severity Index; MCH-SF: Mental Health Continuum - Short Form; NR: Not Reported; OAS: Other as Shamer Scale; PASI: Psoriasis Area and Severity Index; PHQ-4A: Patient Health Questionnaire - Anxiety subscale; PHQ-4D: Patient Health Questionnaire - Depression subscale; PHQ-9: Patient Health Questionnaire - 9 items; POEM: Patient-Oriented Eczema Measure; PPNRS: Peak Pruritus Numerical Rating Scale; PSS: Perceived Stress Scale; SAS: Self-Rating Anxiety Scale; SCS: Self-Compassion Scale; SDS: Self-Rating Depression Scale; VAS: Visual Analogue Scale; VRI: Virtual Reality Intervention.*

**Table S6. Systematic review study quality assessment and limitations detailed results.**

| **Authors** | **Equity Considerations** | **Acceptability and Usability** | **Barriers and Facilitators** | **Risk of Bias** | **Limitations identified by the authors** | **Quality Assessment** |
| --- | --- | --- | --- | --- | --- | --- |
| van Beugen et al., 2016 [20] | Computer/internet access required; no specific equity analysis | Satisfaction 7.6/10; 85% would recommend | Working alliance predicted outcomes; lower baseline severity linked to dropout | Open-label RCT; attrition 26%; moderate risk | High CAU effect; low baseline distress; attrition; mild disease severity | High |
| Domogalla et al., 2021 [24] | No specific equity analysis | 39% used app >20% of the once weekly information logging opportunities | Reminder of chronic disease with frequent app use; telemedicine reducing clinic visits (less healthcare cost and less absence of work) | Open-label RCT, no follow-up | Monocentric design; small study cohort; limited generalizability of the results. | High |
| Fortune et al., 2022 [22] | Smartphones users w/o reading-writing impairment; no specific equity analysis | 78% of n=34 at 12 weeks asked to keep using the app | Improvement in accessibility options; highly convenient and immediately accessible platforms | Selection bias, no follow-up | Limited generalizability of the results; no RCT, no long-term follow-up | Moderate |
| Leibovici et al., 2009 [26] | randomized groups, and analysis shows groups are statistically similar | AVD more accessible | Opportunity to practice on computer before starting levels for users not familiar with computer games | Selection bias; no control group; objective 3 points scale from multiple interviewers; small sample, no follow-up | Small sample; software programs appropriate for different age/sex/cultural background | Moderate |
| Muftin et al., 2022 [22] | No specific equity analysis | 70% found material helpful | Email communication | Selection bias (responding to email of interested); no control group; no comparing the similarity between randomized cohorts; no compilation of how many times patient used material | Majority are young, educated female patients (generalization difficulty); recruiting and treatment all online; lack of measure of psoriasis severity; some patients used anti-anxiety medication | Moderate |
| Zhou et al., 2024 [25] | No specific equity analysis | Online MCBT increase patient compliance and trust in doctors | Economical, beneficial to remote/rural | Open-label RCT, no follow-up | One site in poor socioeconomic thus higher anxiety and depression rate; no research on mental health of chronic disease on minors/adolescents | High |
| Hedman-Lagerlöf et al., 2021 [2021] | No specific equity analysis | High treatment satisfaction from patients | Improves access to treatment; increase stress and worry from having to actually do therapy weekly | Majority female | Patient measured (no medical or nursing measures), | Moderate |
| Kern et al., 2023 [28] | No specific equity analysis | System usability score 66,7% | No therapist guidance | Majority female, small sample size, no ctrl group | Single male participant, small sample size, no ctrl group, season variations on AD | Moderate |
| Kern et al., 2025 [12] | No specific equity analysis | System usability score 66% | Data loss for secondary outcome because patients did not answer all questions | Majority female | Post-intervention data loss for secondary outcomes, measures validity of scales, no data on race/ethnicity | Moderate |
| Clarke et al., 2024 [23] | No specific equity analysis | Positive feedback score to website | Easy to provide and facilitate treatment | Majority female, no ctrl group, small sample size, no follow up | Positive-biased feedback, majority female | Moderate |
| Sengupta et al, 2025 [27] | No specific equity analysis | NR | NR | Very high socioeconomic status with half of participants unemployed with time to complete modules, selection bias, no specific baseline to skin conditions | Heterogenous skin conditions, no qualitative methods to assess subjective experience of participants, no long-term follow-up on effect | Moderate |

*Acronyms: AD: Atopic Dermatitis; AVD: Alternative Video Distraction; CAU: Care As Usual; ctrl: Control; MCBT: Mindfulness-Based Cognitive Therapy; NR: Not Reported; RCT: Randomized Controlled Trial; SUS: System Usability Scale.*
